# Dietary Inflammatory Index and Nutritional Status in Children with Inborn Errors of Metabolism on Protein-Restricted Diets

**DOI:** 10.3390/nu17183010

**Published:** 2025-09-20

**Authors:** Emine Aktaş, Betül Çiçek, Ilyas Okur, Asli İnci, Leyla Tümer

**Affiliations:** 1 Department of Nutrition and Dietetics, Institute of Health Sciences, Erciyes University, Kayseri 38039, Türkiye; 2 Department of Pediatric Nutrition and Metabolic Disorders, Faculty of Medicine, Gazi University, Ankara 06560, Türkiyeaslidinci@gmail.com (A.İ.);; 3 Department of Nutrition and Dietetics, Faculty of Health Sciences, Erciyes University, Kayseri 38260, Türkiye

**Keywords:** metabolic diseases, pediatrics, protein restricted diets

## Abstract

**Background**: The primary treatment for inborn errors of metabolism (IEM) involves restricted intake of natural protein. Inadequate diets can lead to an increased risk of inflammation and susceptibility to infections. The Dietary Inflammatory Index (DII) is used to estimate whether a diet has anti-inflammatory or pro-inflammatory properties. This study aimed to investigate the relationship between the inflammatory index score of natural protein-restricted diets used in medical nutrition therapy for IEM intoxication, the anthropometric measurements and nutritional status of affected children. **Method:** The study included 20 patients (5 organic acidemia, 5 urea cycle disorders, 10 phenylketonuria) and 20 healthy children. Patients followed a natural protein-restricted diet, while the healthy control group maintained their usual dietary habits. Dietary records were collected for both groups, and the DII and macro-micronutrient intakes were calculated. **Result:** DII scores were similar between the patient and control groups. Anthropometric measurements did not differ significantly between the groups. However, carbohydrate and fat intakes were higher in the patient group compared to the control group (*p* < 0.05). Additionally, comparative analyses revealed that vitamin B1, C and E, iron, and magnesium intakes were higher in the patient group than in the control group. **Conclusions:** Children on a natural protein-restricted diet showed growth patterns comparable to their healthy peers. This study demonstrated that nutritional deficiencies can be prevented in amino acid metabolism disorders treated with a natural protein-restricted diet by carefully controlling nutrition with vitamin and mineral-fortified formulas.

## 1. Introduction

Inborn errors of metabolism (IEM) disorders are typically caused by enzymatic defects in carbohydrate, protein, and lipid metabolism. These disorders vary widely in clinical presentation and severity and are usually inherited in an autosomal recessive manner [1,2]. Effective treatments include dietary management, cofactor supplementation, enzyme replacement therapy, and, more recently, gene therapy [3].

Phenylketonuria (PKU), the most common amino acid metabolism disorder, results from a complete or partial deficiency in phenylalanine hydroxylase, the enzyme responsible for converting phenylalanine to tyrosine [4]. Although untreated newborns with PKU appear normal at birth, symptoms emerge within a few months. These may include a musty odor in the skin and urine, fair skin, eczema, seizures, tremors, and hyperactivity [5].

Hereditary tyrosinemia type 1 (HT1) is an autosomal recessive disorder caused by a defect in the tyrosine catabolic pathway. Treatment involves medications that inhibit the production of toxic metabolites and a tyrosine-restricted diet [6].

Homocystinuria is a sulfur metabolism disorder characterized by elevated plasma homocysteine (Hcy) levels, leading to vascular and neurological complications [7]. It is characterized by markedly elevated plasma homocysteine (Hcy) and methionine levels, leading to vascular complications (such as thromboembolism, atherosclerosis, and stroke), ocular manifestations (lens dislocation), skeletal abnormalities (marfanoid habitus, osteoporosis), and neurological impairment (intellectual disability, psychiatric symptoms) Treatment strategies include vitamin B6, vitamin B12, and folate supplementation, along with a me-thionine-restricted diet to lower homocysteine levels. In some cases, betaine therapy is also used to promote the re-methylation of homocysteine to methionine [8].

Isovaleric aciduria (IVA) is caused by a deficiency of isovaleryl-CoA dehydrogenase, propionic aciduria (PA), by a deficiency of propionyl-CoA carboxylase, and methylmalonic aciduria (MMA) by a deficiency of methylmalonyl-CoA mutase [9]. These deficiencies lead to the accumulation of toxic organic acids, causing metabolic acidosis, a key clinical feature of these conditions. Treatment primarily involves restricting natural amino acids, which leads to the accumulation of toxic metabolites. Additionally, long-term management of MMA and PA includes L-carnitine, vitamin B12 (for cobalamin-responsive MMA), and nitrogen-scavenging agents along with diet [10].

Urea cycle disorders (UCDs) are caused by defects in urea cycle enzymes and transport proteins leading to impaired ammonia excretion during protein catabolism. The resulting hyperammonemia is highly neurotoxic. Long-term management includes a natural protein–restricted diet tailored to individual tolerance, supplementation with arginine and/or citrulline (to bypass enzymatic blocks and promote nitrogen excretion), and nitrogen-scavenging medications. In severe cases, liver transplantation may provide a definitive cure [11].

Maple syrup urine disease (MSUD) is caused by a deficiency in the activity of the branched-chain α-ketoacid dehydrogenase (BCKD) complex, promoting the accumulation of the branched-chain amino acids (BCAA) leucine, isoleucine, and valine, as well as their respective α-keto acids. MSUD is an autosomal recessive hereditary metabolic disorder characterized by ketoacidosis, ataxia, coma, and mental and psychomotor retardation. The recommended treatment consists of a high-calorie diet with restricted protein intake and specific formulas containing essential amino acids, except those accumulated in MSUD. This treatment will be maintained throughout life, being adjusted according to the patients’ nutritional needs and BCAA concentration. Because dietary treatment may not be sufficient to prevent neurological damage in MSUD patients, other therapeutic strategies have been studied, including liver transplantation [12].

A protein-restricted diet is essential for the management of IEM. However, excessive restriction can impair growth and disrupt normal physiological processes. To avoid deficiencies, the Food and Agriculture Organization (FAO) and the World Health Organization (WHO) recommend maintaining safe levels of protein intake [2]. In addition, children on natural protein–restricted diets are at increased risk of micronutrient deficiencies, particularly vitamin B12, iron, calcium, zinc, and copper; therefore, careful dietary monitoring and appropriate supplementation are necessary to ensure adequate growth and metabolic stability [13].

The Dietary Inflammatory Index (DII) is used to evaluate the inflammatory potential of a diet. It is calculated by assigning scores to nutrients based on their influence on serum inflammatory cytokines levels. Nutrients that elevate pro-inflammatory markers (e.g., CRP, TNF-α, IL-1β and IL-6) receive a positive coefficient, whereas those that enhance anti-inflammatory markers (e.g., IL-4 and IL-10) receive a negative coefficient. A high DII score indicates a pro-inflammatory diet, whereas a low DII score suggests an anti-inflammatory diet that mitigates inflammation [14,15].

DII has been frequently used in studies investigating the relationship between disease and nutrition, as well as in public health research. However, its use is limited in children with IEM. Since these children follow a restricted diet from birth throughout their lives, their nutrition differs from that of other children. This suggests that DII is different for these children [16,17].

In this study, the relationship between the inflammatory index score of natural protein-restricted diets used in medical nutrition therapy for IEM intoxication and children’s anthropometric measurements and nutritional status was investigated.

## 2. Materials and Methods

The study included 20 patients aged 2 to 18 years who had been on a naturally protein-restricted diet for at least two years. These patients were followed at the Gazi University Pediatric Metabolism Polyclinic. Twenty healthy, free-feeding children were also included in the control group. (Ethics Committee Approval: 528) Informed consent was obtained from participants in both groups. Children younger than 2 years of age were excluded from the study because they are usually formula-fed and there would be no difference in their dietary patterns. Children who refused to participate were excluded from the study. Patients receiving enteral nutrition via a feeding tube were excluded from the study.

A structured questionnaire was administered through face-to-face interviews to collect data on sociodemographic characteristics, dietary habits, and anthropometric measurement.

Food consumption was recorded over three days, including one weekend. Dietary intake was analyzed using the Computer-Assisted Nutrition Program (BEBIS), developed for Turkey. Macronutrients (energy, carbohydrates, protein, fat, fiber), vitamins (Vitamins A, D, E, C, thiamine, niacin, riboflavin), and minerals (calcium, iron, zinc, iodine) were calculated for each child based on their average nutrient intake.

Average nutrient intake was calculated for each child.These nutrient intakes were multiplied by coefficients determined in previous studies to calculate the DII [14]. A negative DII score indicates an anti-inflammatory diet, whereas a positive score indicates a pro-inflammatory diet [14]. The macro and micronutrients contained in the protein supplements used by patients were also included in the DII calculation.

### Statistical Analysis

Descriptive statistics, including mean, standard deviation, median, minimum and maximum values, frequency, and percentages, were used to summarize the data. The normality of variable distributions was assessed using the Kolmogorov–Smirnov and Shapiro–Wilk test.

For quantitative data with a normal distribution, the independent samples t-test was used. The Mann–Whitney U test was used to analyze quantitative data that did not follow a normal distribution. The chi-square test was used for categorical data, and Fisher’s exact test was applied when the chi-square test assumptions were not met. Statistical analyses were performed using SPSS 28.0 software.

## 3. Results

Among the 40 participants, 21 were female and 19 were male. Twenty participants followed no specific diet and were included in the control group, whereas the remaining 20 were in the case group, adhering to a natural protein-restricted diet (Table 1).

Z-scores below −2 SD were classified as underweight, whereas a Z-score above +2 SD were classified as obese. Based on weight Z-scores, 7.5% of the participants were underweight, while 10% were underweight according to body mass index (BMI) Z-scores. The waist-to-hip ratio was within the normal range for 42.5% of participants. Age and gender distributions were similar between the case and control groups. Additionally, no significant differences were observed in anthropometric measurements between the two groups (Table 2). It was observed that the growth curves of the protein-restricted group were similar to their peers.

The Dietary Inflammatory Index (DII) did not differ significantly between the case and control groups. Energy intake was also comparable between the two groups. However, carbohydrate intake was significantly higher in the case group, whereas protein and fat intakes were significantly lower than those the control group (Table 3). Children on a natural protein-restricted diet had significantly lower adequate fat consumption than those in the control group. Although the total fiber intake was similar between the case and control groups, fiber adequacy was significantly higher in the case group.

In comparative analyses, vitamin B1, vitamin C, and vitamin E intakes were significantly higher in the case group than in the control group (Table 4). Regarding vitamin adequacy, vitamin D sufficiency was significantly higher in the case group.

Although iron intake was significantly higher in the case group, iron adequacy did not differ significantly between groups. Magnesium intake was also significantly higher in the case group, whereas iodine intake was significantly lower than in the control group (Table 5).

## 4. Discussion

In this study, the DII was used to assess the inflammatory potential of children’s dietary habits. A high DII score indicates a diet with strong pro-inflammatory potential [18].

An anti-inflammatory diet is characterized by high levels of anti-inflammatory markers and is predominantly composed of vegetables, fruits, fish, and legumes. These foods are rich in unsaturated fatty acids, fiber, vitamin C, and essential micronutrients such as calcium, phosphorus, and magnesium [19]. Although the molecular mechanisms underlying the relationship between nutrients and their anti-inflammatory effects are not fully understood, studies suggest that unsaturated fatty acids, fibers, and vitamins play a role in regulating circulating biomarkers [20].

DII has been frequently used in different age groups and for different diseases. A study investigating the relationship between nutrition and dementia risk in community-dwelling older adults used the DII. In this study, higher DII scores (indicating greater pro-inflammatory dietary potential) were associated with an increased risk of dementia [21].

Numerous studies have found that DII is associated with increased obesity, type 2 diabetes, and cardiovascular risk [22,23].

Pro-inflammatory diets have been associated with a higher risk of severe non-alcoholic fatty liver disease, independent of confounding factors such as metabolic syndrome components [16].

While studies on DII exist in different age groups, studies in children are limited. Since metabolic diseases are rare, there are no studies in this group. Although the overall DII scores were similar between the case and control groups, a higher proportion of participants in the case group followed an anti-inflammatory diet. This may be attributed to the lower lipid intake in the case group, as well as the consumption of including low-protein and specialized formulas, which are enriched with vitamins and minerals. Adequate vitamin and mineral intake may contribute to lower DII scores and an overall anti-inflammatory dietary pattern. Natural protein-restricted diets tailored to patients may have a higher anti-inflammatory effect than free diets.

A pro-inflammatory diet has been linked to childhood overweight and obesity [24]. Higher DII scores have been associated with increased BMI and Z-scores in children, which may be related to the consumption of energy-dense, pro-inflammatory foods such as fast food, cookies, and crackers [25,26]. However, in our study, no significant differences were observed in DII scores or anthropometric measurements between the two groups. This suggests that children on a natural protein-restricted diet may achieve growth patterns similar to their peers when provided with appropriate calorie and protein supplementation.

Long term protein restriction is required for dietary management of inherited disorders of protein metabolism. However, prolonged protein restriction raises concerns about growth, as insufficient protein intake can lead to growth retardation, impaired protein synthesis, and micronutrient deficiencies [27]. Studies on the growth parameters of pediatric IEM patients treated with natural protein-restricted diets remain limited. Our findings suggest that with appropriate dietary management, these patients can achieve growth and development comparable to that of healthy children.

During early childhood, dietary protein intake is closely linked to growth parameters, and micronutrient deficiencies can negatively impact growth [28]. Amino acid (AA) supplements are commonly used to prevent protein deficiencies, but dosage recommendations vary between medical centers, and standardized guidelines remain unclear. Additionally, the micronutrient composition of amino acid supplements differs, necessitating separate supplementation to prevent deficiencies. With the increased practice of fortifying phenylalanine-free L-amino acids with vitamins, minerals, and trace elements (including selenium), fewer cases of biochemical vitamin and mineral deficiencies have been reported in phenylketonuria (PKU) patients. However, concerns have arisen regarding excessive micronutrient intake from high-dose amino acid supplementation [29].

Patients with urea cycle disorders (UCD) and branched-chain amino acid disorders, intakes of calcium, magnesium, potassium, zinc, copper, manganese, iodine, and vitamin B12 have been found to be below recommended levels, highlighting the need for regular micronutrient monitoring in all patients with inherited metabolic disorders treated with natural protein-restricted diets [2].

In our study, the intake of vitamin B1, vitamin C, vitamin E, iron, and magnesium was higher in the case group compared to the control group. This may be attributed to the protein supplements consumed by the case group, which provide an adequate supply of vitamins and minerals, thereby preventing severe deficiencies.

Our study has some limitations. Metabolic disorders are rare, so the number of patients is small; an increase in the number of patients or results from multicenter studies would be statistically more meaningful. In addition, although every child is on a protein-restricted diet, the type of amino acid restricted varies from child to child. The heterogeneity of the group may have affected the results.

Furthermore, since dietary records are based on patient and parent reports, portion sizes may not be precise.

## 5. Conclusions

It is a simple and effective index used in studies linking DII diseases to nutrition. Although studies in children are limited, we used DII in children.

Dietary inflammation indices and other nutritional elements of children who follow a lifelong natural protein-restricted diet and meet their protein needs with special formula are similar to those of healthy children. However, this evidence requires further validation.

## Figures and Tables

**Table 1:** Demographic Characteristics and Anthropometric Measurements.

		Min-Max			Median	Mean. ± ss/*n*-%		
Age (Year)		3.0	-	17.0	9.5	10.5	±	4.9
Gender	Female					21		52.5%
	Male					19		47.5%
Weight (kg)		13.0	-	80.0	36.1	40.3	±	20.1
Weight Z Score		−3.1	-	2.7	0.3	0.3	±	1.4
Weight Z Score	Thin					3		7.5%
	Normal					31		77.5%
	Obese					6		15.0%
Height (cm)		89.0	-	173.0	139.0	140.3	±	25.3
Height Z Score		−1.7	-	3.1	0.4	0.3	±	1.1
Height Z Score	Normal					38		95.0%
	Tall					2		5.0%
BMI		12.6	-	28.7	18.5	19.0	±	4.2
BMI Z Score		−3.5	-	2.6	0.1	0.0	±	1.6
BMI Z Score	Thin					4		10.0%
	Normal					31		77.5%
	Obese					5		12.5%
Waist		25.0	-	98.0	67.5	66.8	±	14.8
Hip		27.0	-	109.0	76.0	77.8	±	17.1
Waist/Hip		0.56	-	1.08	0.86	0.86	±	0.09
Waist/Hip	Normal					17		42.5%
	Over weight					21		52.5%
	Obese					2		5.0%
Diagnosis	Control					20		50.0%
	Phenylketonuria					10		25.0%
	Ornithine aminotransferase deficiency					2		5.0%
	Urea Cycle Disorders					2		5.0%
	Glutaric Aciduria Type I					1		2.5%
	Homocystinuria					2		5.0%
	Methylmalonic Acidemia					1		2.5%
	Maple Syrup Urine Disease					1		2.5%
	Tyrosinemia Type I					1		2.5%

**Table 2:** Comparison of demographic characteristics and anthropometric measurements between case and control groups.

		Control (*n* = 20)				Case (*n* = 20)				*p*	
		Mean. ± ss/*n*-%			Median	Mean. ± ss/*n*-%			Median		
Age (Year)		9.4	±	5.0	8.5	11.5	±	4.7	13.0	0.211	^m^
Gender	Female	8		40.0%		13		65.0%		0.113	^X2^
	Male	12		60.0%		7		35.0%			
Weight (kg)		37.0	±	19.9	32.8	43.6	±	20.2	41.2	0.306	^t^
Weight Z Score		0.44	±	1.33	0.28	0.17	±	1.47	0.35	0.925	^m^
Weight Z Score	Thin	0		0.0%		3		15.0%		0.376	^X2^
	Normal	16		80.0%		15		75.0%			
	Obese	4		20.0%		2		10.0%			
Height (cm)		134.9	±	26.3	135.3	145.7	±	23.8	154.0	0.208	^m^
Height Z Score		0.37	±	0.93	0.52	0.24	±	1.26	0.21	0.717	^t^
Height Z Score	Normal	20		100.0%		18		90.0%		0.487	^X2^
	Tall	0		0.0%		2		10.0%			
BMI		18.6	±	4.2	18.4	19.3	±	4.2	18.5	0.626	^m^
BMI Z Score		0.09	±	1.58	0.11	−0.04	±	1.68	0.12	0.808	^t^
BMI Z Score	Thin	1		5.0%		3		15.0%		0.243	^X2^
	Normal	15		75.0%		16		80.0%			
	Obese	4		20.0%		1		5.0%			
Waist		66.3	±	13.7	67.3	67.4	±	16.1	67.5	0.819	^t^
Hip		76.9	±	14.5	75.5	78.7	±	19.7	78.0	0.754	^t^
Waist/Hip		0.86	±	0.12	0.85	0.86	±	0.06	0.87	0.878	^t^
Waist/Hip	Normal	10		50.0%		7		35.0%		0.337	^X2^
	Over weight	8		40.0%		13		65.0%			
	Obese	2		10.0%		0		0.0%			

^t^ independent sample *t* test/^m^ Mann–Whitney u test/^X2^ Ki-kare test.

**Table 3:** Dietary inflammation index, macronutrient intakes and adequacy of these intakes of the case and control groups.

		Control (*n* = 20)				Case (*n* = 20)				*p*	
		Mean. ± ss/*n*-%			Median	Mean. ± ss/*n*-%			Median		
DII		−1.15	±	1.81	−0.93	−1.92	±	2.78	−1.87	0.099	^m^
Anti-inflammatory		15		75.0%		17		85.0%		0.429	^X2^
Pro-inflammatory		5		25.0%		3		15.0%			
Energy (Kcal)		1837.5	±	483.9	1748.5	1937.6	±	543.7	1839.6	0.685	^m^
Energy Status	Deficiency	2		10.0%		1		5.0%		1.000	^X2^
	Adequate	16		80.0%		17		85.0%			
	Excessive	2		10.0%		2		10.0%			
Carbohydrate (gr)		235.1	±	86.0	219.5	340.2	±	103.5	335.1	0.001	^t^
Carbohydrate Status	Deficiency	7		35.0%		1		5.0%		0.018	^X2^
	Adequate	12		60.0%		14		70.0%			
	Excessive	1		5.0%		5		25.0%			
Carbohydrate %		50.6	±	10.5	49.0	70.1	±	11.0	71.5	0.000	^t^
Carbohydrate % Status	Deficiency	2		10.0%		0		0.0%		0.487	^X2^
	Adequate	18		90.0%		15		75.0%			
	Excessive	0		0.0%		5		25.0%			
Formula Protein (gr)		0.0	±	0.0	0.0	24.2	±	15.3	17.7	0.000	^m^
Natural Protein (gr)		65.2	±	21.2	68.9	28.5	±	23.5	21.7	0.000	^m^
Total Protein (gr)		65.2	±	21.2	68.9	52.8	±	28.3	45.7	0.021	^m^
Protein Status	Deficiency	2		10.0%		3		15.0%		0.633	^X2^
	Adequate	13		65.0%		15		75.0%			
	Excessive	5		25.0%		2		10.0%			
Protein %		14.7	±	4.8	14.0	10.8	±	3.7	10.5	0.006	^t^
Protein % Status	Deficiency	0		0.0%		4		20.0%		0.035	^X2^
	Adequate	9		45.0%		11		55.0%			
	Excessive	11		55.0%		5		25.0%			
Lipid (gr)		75.3	±	39.3	71.4	41.8	±	21.5	36.9	0.001	^m^
Lipid Status	Deficiency	1		5.0%		9		45.0%		0.003	^X2^
	Adequate	14		70.0%		10		50.0%			
	Excessive	5		25.0%		1		5.0%			
Lipid %		34.8	±	10.0	33.0	19.1	±	8.8	18.5	0.002	^t^
Lipid % Status	Deficiency	0		0.0%		7		35.0%		0.004	^X2^
	Adequate	11		55.0%		11		55.0%			
	Excessive	9		45.0%		2		10.0%			
Fiber (gr)		15.8	±	6.3	13.7	18.2	±	6.2	18.9	0.144	^m^
Fiber Status	Deficiency	15		75.0%		8		40.0%		0.025	^X2^
	Excessive	5		25.0%		12		60.0%			

^t^ independent sample t test/^m^ Mann–Whitney u test/^X2^ Ki-kare test.

**Table 4:** Vitamin Intake and Sufficiency of Vitamin Intake in Case and Control Groups.

		Control (*n* = 20)				Case (*n* = 20)				*p*	
		Mean. ± ss/n-%			Median	Mean. ± ss/n-%			Median		
Vitamin A (µg)		765.7	±	443.2	664.4	870.4	±	434.9	773.6	0.213	^m^
Vitamin A Status	Deficiency	1		5.0%		1		5.0%		1.000	^X2^
	Adequate	10		50.0%		10		50.0%			
	Excessive	9		45.0%		9		45.0%			
Vitamin C (mg)		79.1	±	50.2	62.2	165.4	±	105.1	173.2	0.006	^m^
Vitamin C Status	Deficiency	3		15.0%		2		10.0%		0.633	^X2^
	Adequate	4		20.0%		4		20.0%			
	Excessive	13		65.0%		14		70.0%			
Vitamin D (µg)		2.1	±	1.4	1.8	12.8	±	6.5	13.4	0.000	^t^
Vitamin D Status	Deficiency	16		80.0%		3		15.0%		0.000	^X2^
	Adequate	4		20.0%		0		0.0%			
	Excessive	0		0.0%		17		85.0%			
Vitamin E (mg)		10.8	±	6.5	10.3	17.5	±	9.2	13.5	0.018	^m^
Vitamin E Status	Deficiency	7		35.0%		2		10.0%		0.058	^X2^
	Adequate	8		40.0%		8		40.0%			
	Excessive	5		25.0%		10		50.0%			
Vit B_1_/Tiamin (mg)		0.76	±	0.36	0.72	1.45	±	0.99	1.30	0.000	^m^
Vitamin B_1_ Status	Deficiency	5		25.0%		2		10.0%		0.212	^X2^
	Adequate	12		60.0%		7		35.0%			
	Excessive	3		15.0%		11		55.0%			
Vitamin B_2_/Ribofl. (mg)		1.16	±	0.53	1.20	1.63	±	1.42	1.28	0.262	^m^
Vitamin B_2_ Status	Deficiency	2		10.0%		2		10.0%		1.000	^X2^
	Adequate	8		40.0%		8		40.0%			
	Excessive	10		50.0%		10		50.0%			
Vitamin B_3_/Niacin (mg)		11.7	±	5.9	11.9	18.4	±	14.6	12.8	0.126	^m^
Vitamin B_3_ Status	Deficiency	8		40.0%		4		20.0%		0.168	^X2^
	Adequate	10		50.0%		9		45.0%			
	Excessive	2		10.0%		7		35.0%			
Vitamin B_6_/Pirid. (mg)		0.98	±	0.38	0.90	1.81	±	1.84	1.42	0.058	^m^
Vitamin B_6_ Status	Deficiency	2		10.0%		4		20.0%		0.376	^X2^
	Adequate	9		45.0%		6		30.0%			
	Excessive	9		45.0%		10		50.0%			
Vitamin B_12_ (µg)		3.01	±	2.31	2.36	2.81	±	2.11	2.66	0.935	^m^
Vitamin B_12_ Status	Deficiency	4		20.0%		3		15.0%		0.677	^X2^
	Adequate	4		20.0%		9		45.0%			
	Excessive	12		60.0%		8		40.0%			

^t^ independent sample t test/^m^ Mann–Whitney u test/^X2^ Ki-kare test.

**Table 5:** Mineral Intake and Sufficiency of Mineral Intake in Case and Control Groups.

		Control (*n* = 20)				Case (*n* = 20)				*p*	
		Mean. ± ss/*n*-%			Median	Mean. ± ss/*n*-%			Median		
Folate (µg)		213.7	±	97.1	208.3	164.1	±	91.3	151.0	0.094	^m^
Folate Status	Deficiency	5		25.0%		8		40.0%		0.311	^X2^
	Adequate	11		55.0%		8		40.0%			
	Excessive	4		20.0%		4		20.0%			
Iron (mg)		11.2	±	8.6	8.8	15.1	±	6.2	14.4	0.004	^m^
Iron Status	Deficiency	4		20.0%		3		15.0%		0.677	^X2^
	Adequate	12		60.0%		9		45.0%			
	Excessive	4		20.0%		8		40.0%			
Magnesium (mg)		235.2	±	124.3	198.2	298.5	±	130.4	304.2	0.040	^m^
Magnesium Status	Deficiency	5		25.0%		3		15.0%		0.429	^X2^
	Adequate	8		40.0%		12		60.0%			
	Excessive	7		35.0%		5		25.0%			
Zinc (mg)		9.3	±	4.7	8.1	11.9	±	4.3	12.0	0.074	^m^
Zinc Status	Deficiency	2		10.0%		2		10.0%		1.000	^X2^
	Adequate	8		40.0%		7		35.0%			
	Excessive	10		50.0%		11		55.0%			
Calcium (mg)		603.8	±	286.4	559.7	559.2	±	291.8	506.8	0.685	^m^
Calcium Status	Deficiency	9		45.0%		13		65.0%		0.204	^X2^
	Adequate	8		40.0%		5		25.0%			
	Excessive	3		15.0%		2		10.0%			
Phosphorus (mg)		990.0	±	399.8	930.1	1231.6	±	830.2	1027.9	0.248	^t^
Phosphorus Status	Deficiency	4		20.0%		7		35.0%		0.288	^X2^
	Adequate	6		30.0%		6		30.0%			
	Excessive	10		50.0%		7		35.0%			
Iodine (µg)		109.6	±	49.6	102.4	69.7	±	66.0	58.7	0.002	^m^
Iodine Status	Deficiency	5		25.0%		17		85.0%		0.000	^X2^
	Adequate	12		60.0%		2		10.0%			
	Excessive	3		15.0%		1		5.0%			

^t^ independent sample *t* test/^m^ Mann–Whitney u test/^X2^ Ki-kare test.

## Data Availability

The original contributions presented in the study are included in the article; further inquiries can be directed to the corresponding author.

## Abbreviations

The following abbreviations are used in this manuscript:

IEM	Inborn errors of metabolism
DII	Dietary Inflammatory Index
PKU	Phenylketonuria
IVA	Isovaleric aciduria
MMA	Methylmalonic aciduria
PA	Propionic aciduria
UCD	Urea cycle disorders
BMI	Body mass index
OAT	Ornithine aminotransferase deficiency
MSUD	Maple syrup urine disease
